# Optical Spectra Tuning of All-Glass Photonic Bandgap Fiber Infiltrated with Silver Fast-Ion-Conducting Glasses

**DOI:** 10.3390/ma7085735

**Published:** 2014-08-07

**Authors:** Ioannis Konidakis, Stavros Pissadakis

**Affiliations:** Foundation for Research and Technology-Hellas (FORTH), Institute of Electronic Structure and Laser (IESL), P.O. Box 1385, 71110 Heraklion, Greece; E-Mail: pissas@iesl.forth.gr

**Keywords:** photonic crystal fiber, microstructured fiber design, silver phosphate glass, fast ion conducting, clusters, photonic bandgap guidance

## Abstract

Silver iodide metaphosphate glasses of the *x*AgI + (1−*x*)AgPO_3_ family are embedded inside the air capillaries of a commercial silica photonic crystal fiber (PCF) by means of vacuum-assisted infiltration technique. In this paper, we report on tuning the photonic bandgap (PBG) guidance characteristics of the fabricated all-glass photonic bandgap fibers, by varying the composition of the fast-ion-conducting phosphate glass infiltration medium. Doping AgPO_3_ metaphosphate glass with AgI significantly alters the PBG guidance patterns in the examined range between 350 and 1750 nm, as it leads to the introduction of numerous additional transmission stop-bands, while affecting scattering dependant losses. The effect of phosphate glass cooling method during sample fabrication on the transmission behavior of the *x*AgI + (1−*x*)AgPO_3_/PCFs is also considered.

## 1. Introduction

Photonic bandgap (PBG) optical fiber guidance relies on the existence of a refractive index contrast opto-geometrical configuration, where a low refractive index photonic crystal fiber (PCF) core is surrounded by high refractive index strands ordered in a periodic lattice, while rendering the low refractive index fiber core a phase defect of this periodic lattice wherein propagation at discrete frequencies is allowed [1,2,3]. This specific guidance mechanism has been also successfully described by antiresonant reflecting optical waveguide (ARROW) guidance theory, assuming guidance into the PCF core for light in the form of leaky modes into the high refractive index strands. Thus, by tuning the refractive index, absorption loss, and structural/scattering properties of the infiltrated media inside the PCF capillaries, the spectral signature of the PBG-PCF can be easily tuned. On this basis, the infiltration of PCFs’ air capillaries with high refractive index media, such as inorganic glasses, appears promising towards the design of in-fiber light emitting and sensing devices exploiting PBG or modified total internal reflection guidance [4,5,6,7,8]. Recently, the fabrication of an all-glass PBG fiber by vacuum-assisted infiltration of molten silver metaphosphate (AgPO_3_) glass into an air/silica PCF was demonstrated [6,7]. Initially, the effect of AgPO_3_ glass laser photosensitivity on the guiding properties of the composite PCF was investigated [6], whereas, the latter study focuses on shaping the PBG guiding properties of AgPO_3_ glass infiltrated PCFs upon introduction of silver nanoparticles inside the phosphate glass strands via thermal poling processes [7]. Along these lines, the potential exploitation of silver nanoparticles plasmon resonance characteristics attracts substantial interest in terms of the development of in-fiber electrically driven devices and sensors [9,10,11].

In the work presented here, we exploit the advantages of another set of functional glasses as PCF infiltrated medium, as we report on the fabrication of all-glass PBG fiber by infiltration of silver fast-ion-conducting (FIC) glasses of the system *x*AgI + (1−*x*)AgPO_3_ into the PCF capillaries. The choice of infiltrating PCFs with FIC phosphate glasses is prompted by variety of reasons and opens up new prospective for developing new devices but also for studying interesting light propagation effects. Apart from their soft nature (extremely low glass transition temperature, *T*_g_) and low melt viscosities that allow the formation of highly homogeneous glass strands inside the PCFs, silver phosphate FIC glasses provide two additional advantages of great importance. Firstly, they are characterized by ionic conductivities up to 10^−2^ S·cm^−1^ at room temperature, *i.e.*, such superior conductivity values are analogous to that of molten salt electrolytes like KNO_3_ used for electric battery applications, and demonstrate the high charge carrier mobility within the glass matrix [12,13,14,15]. Indicatively, the superior mobility of silver cations can be further exploited for tuning the guiding properties of the composite all-glass FIC/PCF, towards the development of in-fiber electrically driven devices [11]. Secondly, the addition of AgI leads to the formation of conduction pathways and AgI-rich microdomains or clusters which percolate though the existing phosphate glass network [14,16,17]. Such microdomains within the glass strands of the infiltrated PCFs act as centers of scattering [6,7,16], and thus, the concentration of AgI is a powerful tool for tuning the PBG guidance characteristics of the FIC glass infiltrated PCFs [18]. The easy tuning of the spectral position of the transmission permitted bands for such an all-solid PCF can be used for overlapping them with the emission bands of active ions (such as Er, Yb or Nd), and developing lasing or amplification components. In addition, the ability to tune the scattering morphology of high transparency FIC glasses, infiltrated inside PCFs, can be used for triggering other light localization effects, in addition to the original PBG guidance. This can be of great importance since the infiltration of glasses of controlled scattering behavior into the capillaries of PCFs, constituting a photonic bandgap system with spatially confined and controlled statistics disorder, can be used for the realization of random lasing [19] and slow light devices [20]. Based on the above, we hereby report on both the effects of AgI concentration and FIC glass cooling method employed for the glass formation on the transmission patterns of composite *x*AgI + (1−*x*)AgPO_3_-glass/PCF samples, while all samples are also examined by scanning electron microscopy (SEM) in order to verify glass filling quality.

## 2. Experimental

### 2.1. Fabrication of xAgI + (1−x)AgPO_3_/PCF Samples

For the experiments of the present study a commercially available all-silica PCF was used, *i.e.*, LMA-10 drawn by NKT Photonics Ltd. (Birkerod, Denmark). The microstructured LMA-10 fiber has a periodic four-ring lattice of hollow capillaries running through the entire length of the PCF. The average diameter of the capillaries is 2.85 μm as determined by scanning electron microscopy (SEM), whereas the lattice constant is found to be 6.4 μm. Based on the silver metaphosphate AgPO_3_ glass, fast-ion-conducting (FIC) glasses of the system *x*AgI + (1−*x*)AgPO_3_ with *x* = 0.3 and 0.55 were prepared by melting appropriate amounts of AgI, AgNO_3_ and NH_4_H_2_PO_4_ dry powders within an electrical furnace [21,22]. All reactions were carefully monitored until gas evolution ceased. The air-capillaries of the LMA-10 fiber were filled by means of a vacuum-assisted infiltration process which is described in detail elsewhere [6]. For the fibers fabricated here, a temperature of 670 °C was maintained throughout the infiltration process. At this temperature, we achieved filling ratios of ~3.5 cm·h^−1^ for the AgPO_3_ glass and ~9 cm·h^−1^ for the two FIC *x*AgI + (1−*x*)AgPO_3_ glasses, *i.e.*, depending on the melt viscosity. Only two PCF samples were infiltrated from each FIC glass melt as HI gas evolution over subsequent reheating may eventually alter the nominal stoichiometry and local topology of the glasses [22]. A splat sample of the *x*AgI + (1−*x*)AgPO_3_ glass with *x* = 0.55 was sandwiched between two silica plates forming an even layer of ~0.06 mm thickness, kept at a temperature of ~150 °C. Spectrophotometric measurements using a white light profilometer (FR-Basic VIS/NIR, ThetaMetrisis S.A., Athens, Greece) of this sample revealed that its short wavelength absorption band sharply starts at ~450 nm.

After the infiltration stage, two distinct cooling methods of the glass melt were followed, namely fast-cooling (f.c.) and slow-cooling (s.c.). Following the fast-cooling method, the infiltrated fiber was instantly removed from the electrical furnace and exposed to ambient room temperature. In contrast, by following the slow-cooling protocol, after the infiltration period the fiber was immerged from the glass melt with the aid of a micrometric stage; however, it remained within the electrical furnace to sustain a monitored cooling rate of ~5 °C·min^−1^. Scanning electron microscopy (SEM) was employed to examine the infiltration quality of the composite LMA-10 samples studied herein. SEM scans were performed in different cross-sections of the entire infiltrated length of the PFC samples in order to ensure axial homogeneity of the phosphate glass strands.

### 2.2. Optical Spectra Measurements

The transmission spectra of homogenous sections of AgPO_3_ and *x*AgI + (1−*x*)AgPO_3_/LMA-10 samples of 1 cm length were measured by using a microscope objective coupling-in/out set-up and a broadband supercontinuum source (SCS, 350–2000 nm), as described in [6]. Namely, a 25× microscope objective was used to couple light into the solid silica core, whereas a 60× objective collected the near field light from the fiber end face. An iris diaphragm was employed for solely selecting the fiber core guided output light, and reducing the stop-band noise lever originating from the light propagation through the phosphate glass strands of the cladding. Finally, the detected light was coupled through a multimode fiber to an optical spectrum analyzer.

## 3. Results and Discussion

### 3.1. Scanning Electron Microscopy (SEM)

**Figure 1:**
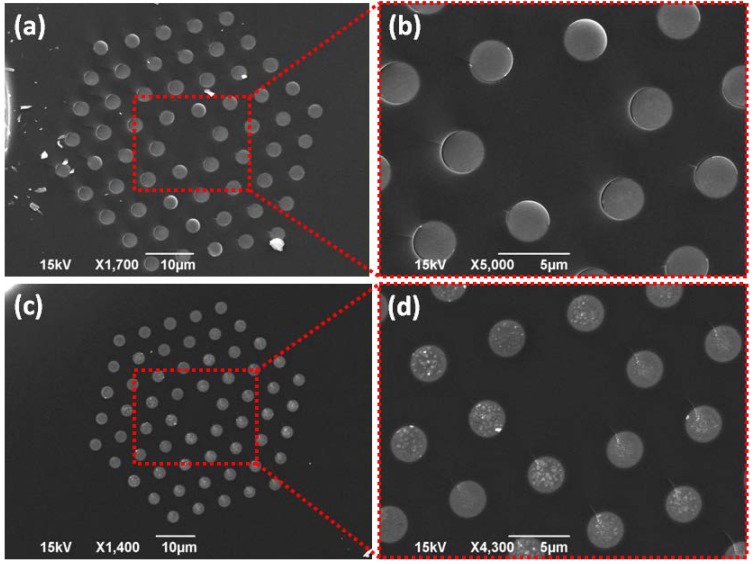
(**a**) SEM scan of a cleaved end face of 0.3AgI + 0.7AgPO_3_/LMA-10 fiber prepared following fast-cooling method; (**b**) Magnified area of panel (**a**); (**c**) SEM scan of the same type of composite fiber prepared following slow-cooling method; (**d**) Magnified area of panel (**c**).

Figure 1a,b show SEM scans of a cleaved cross section of 0.3AgI + 0.7AgPO_3_/LMA-10 fiber prepared following fast-cooling method. The fast-cooled 0.3AgI + 0.7AgPO_3_ FIC glass strands appear to exhibit minimal cluster formation of average size less than 15 nm, as determined by thorough image analysis of the SEM photos by the aid of computing software [23]. Such cluster size is comparable to the size of silver nanoparticles previously reported for the binary AgPO_3_ glass, *i.e.*, prepared under equivalent conditions [7], while resting on the Rayleigh scattering regime. Inspection of Figure 1c,d reveals an average size of ~350 nm for the clusters embedded within the slow-cooled 0.3AgI + 0.7AgPO_3_ glass strands; AgI-nanoparticles of this size rest on the Mie scattering regime, exhibiting angular dependent scattering efficiency.

**Figure 2:**
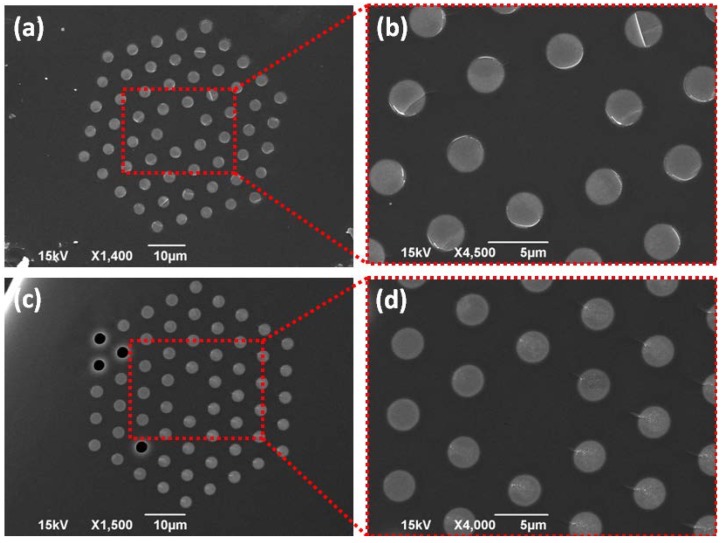
(**a**) SEM scan of a cleaved end face of 0.55AgI + 0.45AgPO_3_/LMA-10 fiber prepared following fast-cooling method; (**b**) Magnified area of panel (**a**); (**c**) SEM scan of the same type of composite fiber prepared following slow-cooling method; (**d**) Magnified area of panel (**c**).

Similar SEM examination was performed on 0.55AgI + 0.45AgPO_3_/LMA-10 samples. Figure 2a,b display details of cross section of samples fabricated following fast-cooling protocol, while corresponding scans for fibers of the slow-cooling protocol are presented in Figure 2c,d. Again, for the fast-cooled FIC glass clusters of negligible size of less than 15 nm are revealed, whereas their size increases to a figure of ~150 nm when the slow-cooling method is followed. Overall, SEM analysis of all fabricated PCFs revealed good quality of FIC phosphate glass strands without hollow inclusions, *i.e.*, bubbles or cracks. We note that SEM scans of the binary metaphosphate glass AgPO_3_/LMA-10 fibers have been extensively presented in previous studies [6,7], and thus, not included herein for the sake of space.

### 3.2. Transmission Spectra of AgPO_3_ and xAgI + (1−x)AgPO_3_/LMA-10 Samples

**Figure 3:**
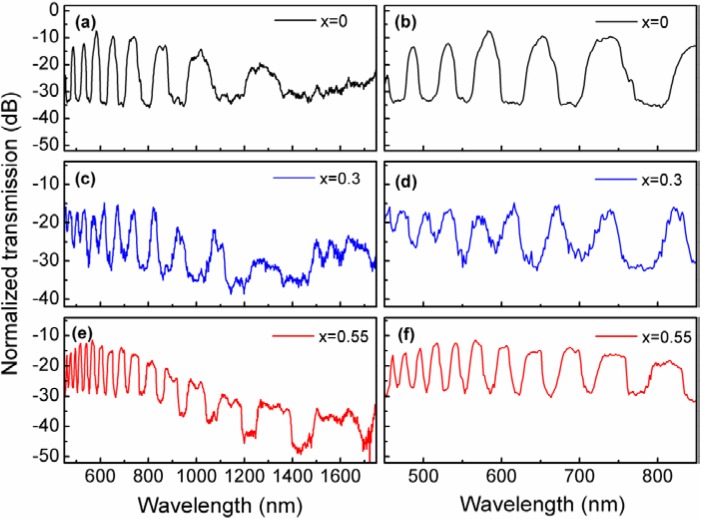
Transmission spectra of 1 cm long *x*AgI + (1−*x*)AgPO_3_/LMA-10 fibers, for *x* = 0, 0.3 and 0.55, prepared following fast-cooling method. Insets on the right present focus on the short wavelength guiding regime between 450 and 850 nm. All spectra are normalized to the corresponding spectrum of the pristine photonic crystal fiber (PCF).

Figure 3 shows typical experimental transmission spectra of 1 cm long *x*AgI + (1−*x*)AgPO_3_/LMA-10 fibers, for *x* = 0, 0.3 and 0.55, prepared following fast-cooling method. All transmission patterns demonstrate photonic bandgap (PBG) guidance with extinction ratios of ~20 dB/cm for the AgI-doped glasses, and ~25 dB·cm^−1^ for the binary AgPO_3_ glass. Due to the higher refractive index of the AgI-doped glasses compared to the undoped, basic silver metaphosphate matrix, further optical bands are inserted into their transmission spectra; as predicted from ARROW theory greater accumulation from those bands appears at the short wavelengths regimes. The reported refractive indexes for the AgI doped glasses with respect to the AgI concentration are 1.79, 1.90 and 2.1, for *x* = 0, 0.3 and 0.55 respectively [24]. Moreover, the transmission losses for the AgI doped glass infiltrated PCFs appear slightly higher than those of the sample for *x* = 0. This is attributed to two main reasons: (a) scattering induced by the favored formation of silver particles for those glass compositions on the interface between the silica PCF and the soft glass inclusion, increases overall attenuation; and (b) the greater the AgI content the higher the absorption losses of the infiltrated glass, shifting bandgap edge to longer wavelengths [25].

**Figure 4:**
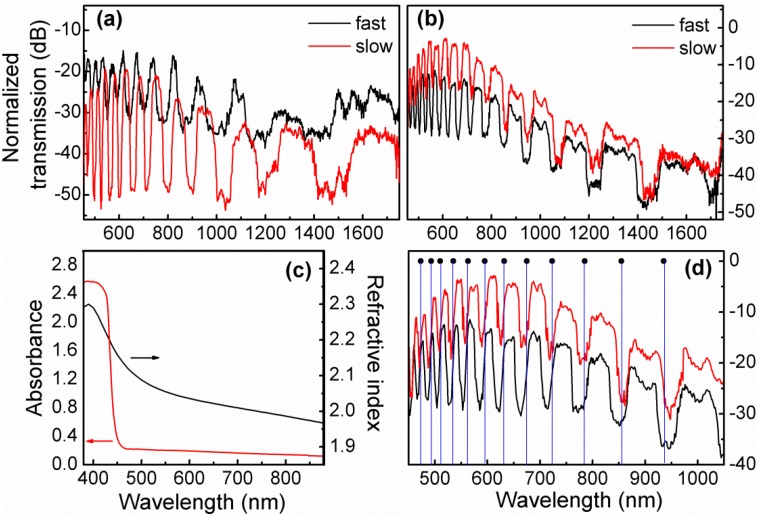
Transmission spectra of 1 cm long *x*AgI + (1−*x*)AgPO_3_/LMA-10 fibers fabricated via fast-cooling and slow-cooling methods, for *x* = 0.3 (**a**), and *x* = 0.55 (**b**), *i.e.*, normalized to the corresponding spectrum of the pristine PCF fiber; (**c**) Absorption and refractive index as obtained from ellipsometry for glass with *x* = 0.55; (**d**) Focus on the short wavelength guiding regime between 450 and 1050 nm for glass with *x* = 0.55, along with ARROW model prediction of the transmission minima [2]. The modal order of the 935 nm notch is *m* = 9.

Then, the effect of the cooling method followed during sample fabrication, *i.e.*, fast-cooling and stop-cooling, on the PBG guidance characteristics of the composite FIC-glass/LMA-10 fibers was investigated. Figure 4 presents transmission patterns for 0.3AgI + 0.7AgPO_3_/LMA-10 and 0.55AgI + 0.45AgPO_3_/LMA-10 fibers. For the first fiber (Figure 4a), where *x* = 0.3, by adopting the slow-cooling protocol, the overall transmission loss increases by as much as ~10 dB·cm^−1^ at specific bands of transmission. Slow cooled PBG-PCF exhibits spectrally shifted transmission stopbands, with respect to the fast-cooled counterpart, indicating that even though glass strands composition of the two infiltrated PCFs is identical, refractive index dispersivity substantially differs between them. There are two significant spectral features characterizing the data of Figure 4a: the first refers to the extinction ratio of the transmission stop-bands that drastically increases from ~15 to ~30 dB·cm^−1^, while the second refers to the full width half maximum (FWHM) broadening of the discrete spectral stopbands up to a factor of 2× for specific bands. Indicatively, the ~837 nm centered band broadens from 16.9 to 32 nm and the ~950 nm centered one from 29.7 to 59 nm. The specific spectral features (extinction ratio increase and broadening of bands) are related with the extensive AgI agglomeration promoted by the slow-cooling process (also verified by SEM investigations presented in Figure 1c,d). 

The spectral data of Figure 4a,b are of particular interest. Generally, phase and amplitude errors introduced in optical lattices used for distributed light scattering or light confinement (extrinsic *etc*.) result in predominant reduction of the scattering efficiency and broadening of the bandwidth of their spectral signature [26,27]. The interesting point observed in the current data refers to the simultaneous spectral broadening of the transmission bandgaps (see Figure 4a,b) and the increase of their extinction ratio (see Figure 4a) for the slow cooled samples; this finding does not agree with the typical cases aforementioned. A plausible explanation can be that the broadening of the bandgaps in the slow cooled FIC infiltrated PCFs comes from their higher refractive index emerging from their thermal history; higher refractive index of the glass strands can justify the spectral shifts of the transmission bands. We speculate that the broadening of the FWHM of the stop-bands for the slow cooling samples is associated with the high refractive index AgI agglomerated microdomains, that affect the light confinement in these FIC glass infiltrated PCFs. Theoretical and experimental studies performed in photonic bandgap slab waveguides and hollow core photonic crystal fibers which exhibit random disorders of their basic unit cell, resulted in broadening of the bandgap transmission spectrum [28] and violation of the Beer-Lambert law that can describe simple propagation loss mechanisms [29].

One should also consider that the large size AgI microdomains exhibit wavelength and angular dependent scattering behavior. Simple Mie scattering calculations for a single 300 nm diameter AgI microdomains assuming a modest 5% increase of refractive index due to agglomeration, revealed broadband peak scattering spanning from 350 to 1000 nm, approximately [30]; also, strong angular scattering occurred for most of those wavelengths for angles up to 60°. These effects can also affect the guiding characteristics of the composite PCF fabricated here. Meanwhile, we are also working further for illustrating the exact physical origin of the spectral broadening of the transmission bands of this PBG guiding PCF.

The effect of the cooling method on the guiding characteristics of the 0.55AgI + 0.45AgPO_3_/LMA-10 fiber is of rather different effect and less prominent compared to the glass with lower AgI concentration. Remarkably, Figure 4b reveals that the slow-cooling protocol almost diminishes transmission losses of the composite PCF in the short wavelength guiding regimes, whereas it reduces loss throughout the entire spectral region. We recall now on the corresponding SEM studies of this composite fiber (Figure 2), where even following the slow-cooling approach, the so-formed AgI microdomains did not exceed the size of ~150 nm, despite the fact of higher AgI content compared to glass with *x* = 0.3. Normally, in such glass systems, the devitrification and tendency for crystallization is expected close to the highest contents of AgI [31]. However, it is believed that glass formation limits within the constraint environment of the fiber capillaries push the crystallization bias to lower contents of AgI, where glasses exhibit higher glass transition temperatures [14,22], and thus, their ability to adapt to external stresses induced from the much harder silica glass upon cooling is reduced. Additionally, among the fibers of this study, the 0.55AgI + 0.45AgPO_3_/LMA-10 sample exhibits the highest refractive index contrast between the core and the infiltrated medium, which results to significant reduction of mode confinement losses. Thus, as long as it remains free of exceptionally large AgI-clusters, as found to be the case herein independently of the cooling protocol (Figure 2), it is expected to exhibit the most optimum transmission behavior, as it is revealed by experimental measurements of the transmission profile (Figure 4b). Among the two cooling protocols, slow-cooling exhibits even better transmission behavior due to the fact that during the elongated cooling period the residual stresses between silica and the extremely soft FIC glass (*T*_g_ = 80 °C for *x* = 0.55) vanish, and thus, reducing the loss sources even further.

The lower dependence of the refractive index of the glass melt upon thermal history conditions for the AgI concentration of *x* = 0.55, allowed us to use the simple ARROW model to verify the transmission minima of the PBG composite optical fiber (see Figure 4d). Thus, the interpretation of the spectral data of Figure 4b using antiguiding ARROW Equation [2], well predicted high loss spectral points, while based on refractive index measurements obtained ellipsometrically for the spectral range between 400 and 1000 nm (shown in Figure 4c), for a thin glass sample prepared by splat quenching technique. As it is appeared in Figure 4d the agreement of the loss maxima ARROW model data refer to both fast and slow cooling protocols applied in the infiltrated fibers of this specific composition.

Finally, it is worth noting, that for the case of the binary metaphosphate AgPO_3_/LMA-10 samples no difference in the transmission profiles was obtained between the two cooling protocols, *i.e.*, fast-cooling and slow-cooling. Moreover, SEM investigations (not shown here) revealed similar quality of AgPO_3_ glass strands within all LMA-10 studied herein, independently of the cooling method followed during sample fabrication. Such findings are explained in terms of the lower tendency for crystallization and phase separation within the stable silver metaphosphate glass AgPO_3_ compared to that within AgI-doped FIC glasses. Therefore, in the case of AgPO_3_ samples, even following the slow cooling protocol does not result to any significant formation of microdomains that will be capable of altering the optical properties of the phosphate glass, and thus, the guiding properties of the composite AgPO_3_/LMA-10 PBG fibers. This observation is in total agreement with what has been found in an earlier study [7], where sole annealing treatment of AgPO_3_ glass-infiltrated PCFs showed no alteration on their guiding properties.

## 4. Conclusions

In conclusion, the transmission characteristics of composite *x*AgI + (1−*x*)AgPO_3_/silica photonic bandgap (PBG) fibers were tuned by varying AgI concentration, as well as, following different cooling methods during sample fabrication. As expected, introduction of AgI within the phosphate glass infiltration medium induces additional transmission stop-bands in the PBG spectra profiles of the fibers, along with spectral allocations attributed to the variation of the refractive index contrast between the silica core and the phosphate glass strands. For the lower AgI content (*x* = 0.3), significant differences were observed among the two cooling protocols employed herein, as slow-cooling resulted to the formation of large AgI microdomains within the phosphate glass, which act as centers of scattering and enhance transmission losses. For high AgI content (*x* = 0.55), crystallization within the fiber capillaries was considerably suppressed, even when adopting the slow-cooling protocol, leading to fibers with minimum losses and optimum PBG transmission characteristics. Ongoing work, involves the exploitation of the enhanced charge carrier mobility within fast-ion-conducting glasses, towards tuning the PBG guidance properties of such type of composite fibers upon application of external voltage on the fiber end faces. Also, the realization of fiber waveguiding geometries based on those silver fast-ion-conducting glasses, while utilizing both periodic and highly disordered structures [32] is currently under investigation.
